# Effectiveness of Homologous and Heterologous COVID-19 Booster Doses Following 1 Ad.26.COV2.S (Janssen [Johnson & Johnson]) Vaccine Dose Against COVID-19–Associated Emergency Department and Urgent Care Encounters and Hospitalizations Among Adults — VISION Network, 10 States, December 2021–March 2022

**DOI:** 10.15585/mmwr.mm7113e2

**Published:** 2022-04-01

**Authors:** Karthik Natarajan, Namrata Prasad, Kristin Dascomb, Stephanie A. Irving, Duck-Hye Yang, Manjusha Gaglani, Nicola P. Klein, Malini B. DeSilva, Toan C. Ong, Shaun J. Grannis, Edward Stenehjem, Ruth Link-Gelles, Elizabeth A. Rowley, Allison L. Naleway, Jungmi Han, Chandni Raiyani, Gabriela Vazquez Benitez, Suchitra Rao, Ned Lewis, William F. Fadel, Nancy Grisel, Eric P. Griggs, Margaret M. Dunne, Melissa S. Stockwell, Mufaddal Mamawala, Charlene McEvoy, Michelle A. Barron, Kristin Goddard, Nimish R. Valvi, Julie Arndorfer, Palak Patel, Patrick K Mitchell, Michael Smith, Anupam B. Kharbanda, Bruce Fireman, Peter J. Embi, Monica Dickerson, Jonathan M. Davis, Ousseny Zerbo, Alexandra F. Dalton, Mehiret H. Wondimu, Eduardo Azziz-Baumgartner, Catherine H. Bozio, Sue Reynolds, Jill Ferdinands, Jeremiah Williams, Stephanie J. Schrag, Jennifer R. Verani, Sarah Ball, Mark G. Thompson, Brian E. Dixon

**Affiliations:** ^1^Department of Biomedical Informatics, Columbia University Irving Medical Center, New York, New York; ^2^NewYork-Presbyterian Hospital, New York, New York; ^3^CDC COVID-19 Emergency Response Team; ^4^Epidemic Intelligence Service, CDC; ^5^Division of Infectious Diseases and Clinical Epidemiology, Intermountain Healthcare, Salt Lake City, Utah; ^6^Center for Health Research, Kaiser Permanente Northwest, Portland, Oregon; ^7^Westat, Rockville, Maryland; ^8^Baylor Scott & White Health, Temple, Texas; ^9^Texas A&M University College of Medicine, Temple, Texas; ^10^Kaiser Permanente Vaccine Study Center, Kaiser Permanente Northern California Division of Research, Oakland, California; ^11^HealthPartners Institute, Minneapolis, Minnesota; ^12^School of Medicine, University of Colorado Anschutz Medical Campus, Aurora, Colorado; ^13^Center for Biomedical Informatics, Regenstrief Institute, Indianapolis, Indiana; ^14^Indiana University School of Medicine, Indianapolis, Indiana; ^15^Fairbanks School of Public Health, Indiana University, Indianapolis, Indiana; ^16^Division of Child and Adolescent Health, Department of Pediatrics, Columbia University Vagelos College of Physicians and Surgeons, New York, New York; ^17^Department of Population and Family Health, Columbia University Mailman School of Public Health, New York, New York; ^18^Children's Minnesota, Minneapolis, Minnesota; ^19^Vanderbilt University Medical Center, Nashville, Tennessee.

CDC recommends that all persons aged ≥18 years receive a single COVID-19 vaccine booster dose ≥2 months after receipt of an Ad.26.COV2.S (Janssen [Johnson & Johnson]) adenovirus vector-based primary series vaccine; a heterologous COVID-19 mRNA vaccine is preferred over a homologous (matching) Janssen vaccine for booster vaccination. This recommendation was made in light of the risks for rare but serious adverse events following receipt of a Janssen vaccine, including thrombosis with thrombocytopenia syndrome and Guillain-Barré syndrome^†^ (*1*), and clinical trial data indicating similar or higher neutralizing antibody response following heterologous boosting compared with homologous boosting (*2*). Data on real-world vaccine effectiveness (VE) of different booster strategies following a primary Janssen vaccine dose are limited, particularly during the period of Omicron variant predominance. The VISION Network^§^ determined real-world VE of 1 Janssen vaccine dose and 2 alternative booster dose strategies: 1) a homologous booster (i.e., 2 Janssen doses) and 2) a heterologous mRNA booster (i.e., 1 Janssen dose/1 mRNA dose). In addition, VE of these booster strategies was compared with VE of a homologous booster following mRNA primary series vaccination (i.e., 3 mRNA doses). The study examined 80,287 emergency department/urgent care (ED/UC) visits^¶^ and 25,244 hospitalizations across 10 states during December 16, 2021–March 7, 2022, when Omicron was the predominant circulating variant.** VE against laboratory-confirmed COVID-19–associated ED/UC encounters was 24% after 1 Janssen dose, 54% after 2 Janssen doses, 79% after 1 Janssen/1 mRNA dose, and 83% after 3 mRNA doses. VE for the same vaccination strategies against laboratory-confirmed COVID-19–associated hospitalizations were 31%, 67%, 78%, and 90%, respectively. All booster strategies provided higher protection than a single Janssen dose against ED/UC visits and hospitalizations during Omicron variant predominance. Vaccination with 1 Janssen/1 mRNA dose provided higher protection than did 2 Janssen doses against COVID-19–associated ED/UC visits and was comparable to protection provided by 3 mRNA doses during the first 120 days after a booster dose. However, 3 mRNA doses provided higher protection against COVID-19–associated hospitalizations than did other booster strategies during the same time interval since booster dose. All adults who have received mRNA vaccines for their COVID-19 primary series vaccination should receive an mRNA booster dose when eligible. Adults who received a primary Janssen vaccine dose should preferentially receive a heterologous mRNA vaccine booster dose ≥2 months later, or a homologous Janssen vaccine booster dose if mRNA vaccine is contraindicated or unavailable. Further investigation of the durability of protection afforded by different booster strategies is warranted.

VISION Network methods have been previously published (*3*). Across 306 ED/UC clinics and 164 hospitals from 10 states, all medical encounters among adults aged ≥18 years with a COVID-19–like illness diagnosis^††^ who had received molecular testing (primarily with reverse transcription–polymerase chain reaction) for SARS-CoV-2 during the 14 days before through 72 hours after the medical encounter were considered eligible. The study period began on the earliest day the Omicron variant accounted for ≥50% of sequenced isolates at each site based on state and national surveillance data (state range = December 16–26, 2021). Vaccination status was categorized based on number and type of vaccine doses received (1 Janssen dose, 2 Janssen doses, 1 Janssen/1 mRNA dose, and 3 mRNA doses^§§^). Patients with no record of vaccination were considered unvaccinated. Because a booster dose following a primary Janssen dose was recommended on October 15, 2021, to ensure accurate comparisons across booster strategies, patients vaccinated with a booster dose >120 days before the index date^¶¶^ were excluded. In addition, patients were excluded if they 1) received only 1 or 2 primary mRNA vaccine doses or >3 mRNA vaccine doses, or received >2 mRNA doses following a primary Janssen dose; 2) received the first Janssen dose 1–13 days earlier or a booster dose 1–6 days earlier; or 3) received a booster dose following a primary Janssen dose earlier than the recommended interval (<2 months after dose 1) or an mRNA booster dose earlier than the recommended interval (<5 months after dose 2).***

Using a test-negative design, investigators estimated VE by comparing the odds of a positive SARS-CoV-2 test result between vaccinated and unvaccinated patients using multivariable logistic regression models (*3*,*4*). Models were adjusted using inverse propensity to be vaccinated weights (calculated separately for each VE estimate) and with age, calendar week of index date, geographic area, local virus circulation (percentage of SARS-CoV-2–positive results from testing within the counties surrounding the facility on the date of the encounter), patient comorbidities including immunocompromise^†††^ (*4*), and factors not balanced by propensity to be vaccinated included as covariates.^§§§^ A statistically significant difference was indicated by nonoverlapping 95% CIs or standardized mean or proportion differences ≥0.2, indicating nonnegligible difference in distributions of vaccination or infection status. All statistical analyses were conducted using R software (version 4.1.2; R Foundation). This study was reviewed and approved by the institutional review boards at participating sites or under a reliance agreement with the Westat, Inc. institutional review board.^¶¶¶^

The study included 80,287 encounters among patients with COVID-19–like illness seeking care at ED/UC facilities (Table 1); 64.8% were unvaccinated, 5.6% had received 1 Janssen dose, 0.6% had received 2 Janssen doses, 1.6% had received 1 Jansen/1 mRNA dose, and 27.4% had received 3 mRNA doses. Among booster strategies, the median interval between receipt of the most recent dose and the ED/UC encounter ranged from 49 to 59 days.

**TABLE 1:** Characteristics of emergency department and urgent care encounters among adults with COVID-19–like illness,* by COVID-19 vaccination status^†^ and SARS-CoV-2 test result — 10 states, December 2021–March 2022^§^

Characteristic	Total no. (column %)	No. (row %)					SMD^¶^	No. (row %)	SMD^¶^
		Unvaccinated	1 Janssen dose (≥14 days)	2 Janssen doses (7–120 days)	1 Janssen/1 mRNA dose (7–120 days)	3 mRNA doses (7–120 days)		Positive SARS-CoV-2 test result	
**All ED/UC events**	**80,287 (100.0)**	**52,025 (64.8)**	**4,514 (5.6)**	**467 (0.6)**	**1,271 (1.6)**	**22,010 (27.4)**	**—**	**28,127 (35.0)**	**—**
**Month and year**									
Dec 2021	**17,474 (21.8)**	12,431 (71.1)	1,038 (5.9)	60 (0.3)	200 (1.1)	3,745 (21.4)	0.34	5,785 (33.1)	0.48
Jan 2022	**45,444 (56.6)**	30,812 (67.8)	2,620 (5.8)	242 (0.5)	654 (1.4)	11,116 (24.5)		19,358 (42.6)	
Feb 2022	**16,592 (20.7)**	8,625 (52.0)	806 (4.9)	157 (0.9)	384 (2.3)	6,620 (39.9)		2,953 (17.8)	
Mar 2022	**777 (1.0)**	157 (20.2)	50 (6.4)	8 (1.0)	33 (4.2)	529 (68.1)		31 (4.0)	
**Site**									
Baylor Scott & White Health	**22,536 (28.1)**	18,806 (83.4)	1,068 (4.7)	41 (0.2)	166 (0.7)	2,455 (10.9)	0.89	10,483 (46.5)	0.39
Columbia University**	**1,627 (2.0)**	1,201 (73.8)	70 (4.3)	8 (0.5)	20 (1.2)	328 (20.2)		453 (27.8)	
HealthPartners**	**404 (0.5)**	194 (48.0)	36 (8.9)	3 (0.7)	15 (3.7)	156 (38.6)		156 (38.6)	
Intermountain Healthcare	**18,469 (23.0)**	10,657 (57.7)	1,227 (6.6)	117 (0.6)	427 (2.3)	6,041 (32.7)		5,198 (28.1)	
Kaiser Permanente Northern California	**13,958 (17.4)**	4,366 (31.3)	970 (6.9)	192 (1.4)	387 (2.8)	8,043 (57.6)		3,200 (22.9)	
Kaiser Permanente Northwest	**5,448 (6.8)**	2,729 (50.1)	370 (6.8)	53 (1.0)	112 (2.1)	2,184 (40.1)		1,954 (35.9)	
Regenstrief Institute	**10,975 (13.7)**	8,443 (76.9)	500 (4.6)	42 (0.4)	117 (1.1)	1,873 (17.1)		3,954 (36.0)	
University of Colorado	**6,870 (8.6)**	5,629 (81.9)	273 (4.0)	11 (0.2)	27 (0.4)	930 (13.5)		2,729 (39.7)	
**Age group, yrs**									
18–44	**37,204 (46.3)**	29,740 (79.9)	1,836 (4.9)	68 (0.2)	373 (1.0)	5,187 (13.9)	0.69	14,290 (38.4)	0.2
45–64	**21,457 (26.7)**	12,951 (60.4)	1,623 (7.6)	207 (1.0)	543 (2.5)	6,133 (28.6)		7,752 (36.1)	
65–74	**10,047 (12.5)**	4,789 (47.7)	556 (5.5)	109 (1.1)	181 (1.8)	4,412 (43.9)		3,029 (30.1)	
75–84	**7,392 (9.2)**	3,064 (41.5)	332 (4.5)	61 (0.8)	113 (1.5)	3,822 (51.7)		2,088 (28.2)	
≥85	**4,187 (5.2)**	1,481 (35.4)	167 (4.0)	22 (0.5)	61 (1.5)	2,456 (58.7)		968 (23.1)	
**Sex**									
Male	**33,623 (41.9)**	22,216 (66.1)	2,032 (6.0)	206 (0.6)	519 (1.5)	8,650 (25.7)	0.05	12,313 (36.6)	0.06
Female	**46,644 (58.1)**	29,792 (63.9)	2,481 (5.3)	261 (0.6)	752 (1.6)	13,358 (28.6)		15,807 (33.9)	
Other/Unknown	**20 (—)**	17 (85.0)	1 (5.0)	0 (—)	0 (—)	2 (10.0)		7 (35.0)	
**Race/Ethnicity**									
White, non-Hispanic	**47,305 (58.9)**	28,998 (61.3)	2,890 (6.1)	276 (0.6)	795 (1.7)	14,346 (30.3)	0.29	14,814 (31.3)	0.23
Hispanic	**13,951 (17.4)**	9,836 (70.5)	661 (4.7)	77 (0.6)	215 (1.5)	3,162 (22.7)		5,544 (39.7)	
Black, non-Hispanic	**10,365 (12.9)**	8,185 (79.0)	517 (5.0)	49 (0.5)	117 (1.1)	1,497 (14.4)		4,623 (44.6)	
Other, non-Hispanic	**5,555 (6.9)**	2,738 (49.3)	285 (5.1)	55 (1.0)	107 (1.9)	2,370 (42.7)		1,769 (31.8)	
Unknown^††^	**3,111 (3.9)**	2,268 (72.9)	161 (5.2)	10 (0.3)	37 (1.2)	635 (20.4)		1,377 (44.3)	
**Underlying respiratory condition at discharge^§§^**									
Chronic respiratory condition	**13,761 (17.1)**	8,448 (61.4)	859 (6.2)	107 (0.8)	241 (1.8)	4,106 (29.8)	0.09	4,516 (32.8)	0.04
None	**66,526 (82.9)**	43,577 (65.5)	3,655 (5.5)	360 (0.5)	1,030 (1.5)	17,904 (26.9)		23,611 (35.5)	
**Underlying nonrespiratory condition at discharge** ^¶¶^									
Chronic nonrespiratory condition	**22,917 (28.5)**	13,466 (58.8)	1,417 (6.2)	177 (0.8)	448 (2.0)	7,409 (32.3)	0.19	6,953 (30.3)	0.13
None	**57,370 (71.5)**	38,559 (67.2)	3,097 (5.4)	290 (0.5)	823 (1.4)	14,601 (25.5)		21,174 (36.9)	
Any likely immunocompromise status***									
Yes	**3,399 (4.2)**	1,968 (57.9)	228 (6.7)	29 (0.9)	96 (2.8)	1,078 (31.7)	0.1	996 (29.3)	0.05
No	**76,888 (95.8)**	50,057 (65.1)	4,286 (5.6)	438 (0.6)	1,175 (1.5)	20,932 (27.2)		27,131 (35.3)	
**No. of days from most recent dose to index date, median (IQR)**	—	—	262 (196–293)	59 (34–80)	49 (29–70)	57 (35–77)	—	—	—

**Abbreviations**: ED = emergency department; ICD-9 = *International Classification of Diseases, Ninth Revision*; ICD-10 = *International Classification of Diseases, Tenth Revision*; SMD = standardized mean or proportion difference; UC = urgent care.

* Medical events with a discharge code consistent with COVID-19–like illness were included. COVID-19–like illness diagnoses included acute respiratory illness (e.g., COVID-19, respiratory failure, or pneumonia) or related signs or symptoms (e.g., cough, fever, dyspnea, vomiting, or diarrhea) using ICD-9 and ICD-10 diagnosis codes. Clinician-ordered molecular assays (e.g., real-time reverse transcription–polymerase chain reaction) for SARS-CoV-2 infection occurring ≤14 days before to <72 hours after admission were included.

^†^ Vaccination status was categorized based on number and type of vaccine doses received before the medical event index date, which was the date of respiratory specimen collection associated with the most recent positive or negative SARS-CoV-2 test result before the medical event or the admission date if testing only occurred after admission. A primary Janssen vaccine dose was defined as 1 Janssen dose; a homologous booster dose following a primary Janssen dose was defined as 2 Janssen doses; a heterologous booster dose following a primary Janssen dose was defined as 1 Janssen/1 mRNA dose; a homologous booster dose following a primary mRNA series vaccination was defined as 3 mRNA doses.

^§^ Partners contributing data on medical events and estimated dates of Omicron variant predominance were in California (December 21), Colorado (December 19), Indiana (December 26), Minnesota and Wisconsin (December 25), New York (December 18), Oregon (December 24), Texas (December 16), Utah (December 24), and Washington (December 24).

^¶^ An absolute SMD ≥0.20 indicates a nonnegligible difference in variable distributions between medical events for vaccinated versus unvaccinated patients and for positive versus negative test results. When calculating SMDs for differences in characteristics across COVID-19 vaccination status, investigators calculated SMD as the average of the absolute value of the SMD for unvaccinated versus each vaccination status category individually (1 Janssen, 2 Janssen, 1 Janssen/1 mRNA, and 3 mRNA doses). All SMDs are reported as the absolute SMD.

** ED data at Columbia University Irving Medical Center and HealthPartners exclude encounters that were transferred to an in-network hospital.

^††^ Unknown race/ethnicity includes Asian, Native Hawaiian or other Pacific islander, American Indian or Alaska Native, other not listed, and multiple races.

^§§^ Underlying respiratory condition at discharge was defined as the presence of ICD-9 and ICD-10 discharge codes for asthma, chronic obstructive pulmonary disease, or other lung disease.

^¶¶^ Underlying nonrespiratory condition at discharge was defined as the presence of ICD-9 and ICD-10 discharge codes for heart failure, ischemic heart disease, hypertension, other heart disease, stroke, other cerebrovascular disease, diabetes type I or II, other diabetes, metabolic disease, clinical obesity, clinically underweight, renal disease, liver disease, blood disorder, immunosuppression, organ transplant, cancer, dementia, neurologic disorder, musculoskeletal disorder, or Down syndrome.

*** Immunocompromise status was defined as the presence of ICD-9 and ICD-10 discharge codes for solid malignancy, hematologic malignancy, rheumatologic or inflammatory disorder, other intrinsic immune condition or immunodeficiency, or organ or stem cell transplant.

Overall, VE against laboratory-confirmed COVID-19–associated ED/UC encounters was significantly higher among patients who had received any booster dose (range = 54%–83%) compared with those who had received only 1 Janssen dose (24%) (Table 2). Among booster strategies, VE against laboratory-confirmed COVID-19–associated ED/UC encounters was significantly higher among patients who had received 1 Janssen/1 mRNA (79%) or 3 mRNA doses (83%) than among patients who had received 2 Janssen doses (54%).

**TABLE 2:** Vaccine effectiveness* of 1 primary Janssen vaccine dose, homologous and heterologous boosters following primary Janssen vaccination, and 3 mRNA COVID-19 vaccine doses^†^ against laboratory-confirmed COVID-19–associated emergency department and urgent care encounters and hospitalizations among adults aged ≥18 years^§^ — VISION Network, 10 states, December 2021–March 2022^¶^

Medical event, vaccination status (days since most recent dose)	Total	Positive SARS-CoV-2 result, no. (%)	VE %* (95% CI)
**ED/UC events (N = 80,287)**			
Unvaccinated (Ref)	**52,025**	23,560 (45.3)	Ref
1 Janssen dose ≥14 days earlier (median = 262 days [range = 196–293])	**4,514**	1,652 (36.6)	24 (18–29)
2 Janssen doses (7–120 days)	**467**	135 (28.9)	54 (43–63)
1 Janssen/1 mRNA dose (7–120 days)	**1,271**	166 (13.1)	79 (74–82)
3 mRNA doses (7–120 days)	**22,010**	2,614 (11.9)	83 (82–84)
**Hospitalizations (N = 25,244)**			
Unvaccinated (Ref)	**15,424**	7,271 (47.1)	Ref
1 Janssen dose ≥14 days earlier (median = 264 days [range = 199–294])	**1,451**	518 (35.7)	31 (21–40)
2 Janssen doses (7–120 days)	**164**	47 (28.7)	67 (52–77)
1 Janssen/1 mRNA dose (7–120 days)	**373**	59 (15.8)	78 (70–84)
3 mRNA doses (7–120 days)	**7,832**	775 (9.9)	90 (88–91)

**Abbreviations**: ED = emergency department; UC = urgent care; Ref = referent group; VE = vaccine effectiveness.

* VE was calculated as [1 − odds ratio] x 100%. Odds ratios were estimated using multivariable logistic regression. Models were adjusted using inverse propensity to be vaccinated (weights calculated separately for each VE estimate) and with age, calendar week of index date, geographic area, local virus circulation (percentage of SARS-CoV-2–positive results from testing within the counties surrounding the facility on the date of the encounter), patient comorbidities including immunocompromise, and factors not balanced by propensity to be vaccinated included as covariates. Of the variables included in the propensity score, previous SARS-CoV-2 testing and test positivity were not balanced after applying inverse propensity weights and thus were added to covariates included in the adjusted VE model.

^†^ Vaccination status was categorized based on number and type of vaccine doses received before the medical event index date, which was the date of respiratory specimen collection associated with the most recent positive or negative SARS-CoV-2 test result before the medical event or the admission date if testing only occurred after admission. A primary Janssen dose was defined as 1 Janssen dose; a homologous booster dose following a primary Janssen dose was defined as 2 Janssen doses; a heterologous booster dose following a primary Janssen dose was defined as 1 Janssen/1 mRNA dose; a homologous booster dose following a primary mRNA series vaccination was defined as 3 mRNA doses.

^§^ Medical events with a discharge code consistent with COVID-19–like illness were included. COVID-19–like illness diagnoses included acute respiratory illness (e.g., COVID-19, respiratory failure, or pneumonia) or related signs or symptoms (e.g., cough, fever, dyspnea, vomiting, or diarrhea) using ICD-9 and ICD-10 diagnosis codes. Clinician-ordered molecular assays (e.g., real-time reverse transcription–polymerase chain reaction) for SARS-CoV-2 infection occurring ≤14 days before to <72 hours after admission were included.

^¶^ Partners contributing data on medical events and estimated dates of Omicron predominance were in California (December 21), Colorado (December 19), Indiana (December 26), Minnesota and Wisconsin (December 25), New York (December 18), Oregon (December 24), Texas (December 16), Utah (December 24), and Washington (December 24).

The study included 25,244 hospitalizations among patients with COVID-19–like illness (Table 3); 61.1% were unvaccinated, 5.7% had received 1 Janssen dose, 0.6% had received 2 Janssen doses, 1.5% had received 1 Janssen/1 mRNA dose, and 31.0% had received 3 mRNA doses. Among booster strategies, the median interval between receipt of the most recent dose and hospitalization ranged from 48 to 59 days.

**TABLE 3:** Characteristics of hospitalizations among adults with COVID-19–like illness,* by COVID-19 vaccination status^†^ and SARS-CoV-2 test result — 10 states, December 2021– March 2022^§^

Characteristic	Total no. (column %)	No. (row %)					SMD^¶^	No. (row %)	SMD^¶^
		Unvaccinated	1 Janssen dose (≥14 days)	2 Janssen doses (7–120 days)	1 Janssen/1 mRNA dose (7–120 days)	3 mRNA doses (7–120 days)		Positive SARS-CoV-2 test result	
**All hospitalizations**	**25,244 (100.0)**	**15,424 (61.1)**	**1,451 (5.7)**	**164 (0.6)**	**373 (1.5)**	**7,832 (31.0)**	**—**	**8,670 (34.3)**	**—**
**Month and year**									
Dec 2021	**4,728 (18.7)**	3,048 (64.5)	308 (6.5)	29 (0.6)	46 (1.0)	1,297 (27.4)	0.21	1,370 (29.0)	0.41
Jan 2022	**15,067 (59.7)**	9,631 (63.9)	875 (5.8)	97 (0.6)	206 (1.4)	4,258 (28.3)		6,208 (41.2)	
Feb 2022	**5,438 (21.5)**	2,744 (50.5)	266 (4.9)	38 (0.7)	120 (2.2)	2,270 (41.7)		1,092 (20.1)	
Mar 2022	**11 (—)**	1 (9.1)	2 (18.2)	0 (—)	1 (9.1)	7 (63.6)		0 (—)	
**Site**									
Baylor Scott & White Health	**6,777 (26.8)**	5,198 (76.7)	390 (5.8)	15 (0.2)	77 (1.1)	1,097 (16.2)	0.77	2,523 (37.2)	0.18
Columbia University	**894 (3.5)**	579 (64.8)	65 (7.3)	8 (0.9)	16 (1.8)	226 (25.3)		354 (39.6)	
HealthPartners	**38 (0.2)**	9 (23.7)	5 (13.2)	0 (—)	1 (2.6)	23 (60.5)		9 (23.7)	
Intermountain Healthcare	**2,408 (9.5)**	1,288 (53.5)	133 (5.5)	20 (0.8)	57 (2.4)	910 (37.8)		730 (30.3)	
Kaiser Permanente Northern California	**5,460 (21.6)**	1,791 (32.8)	364 (6.7)	78 (1.4)	138 (2.5)	3,089 (56.6)		1,621 (29.7)	
Kaiser Permanente Northwest	**932 (3.7)**	522 (56.0)	59 (6.3)	11 (1.2)	23 (2.5)	317 (34.0)		264 (28.3)	
Regenstrief Institute	**6,272 (24.8)**	4,320 (68.9)	267 (4.3)	19 (0.3)	48 (0.8)	1,618 (25.8)		2,407 (38.4)	
University of Colorado	**2,463 (9.8)**	1,717 (69.7)	168 (6.8)	13 (0.5)	13 (0.5)	552 (22.4)		762 (30.9)	
**Age group, yrs**									
18–44	**3,976 (15.8)**	3,241 (81.5)	203 (5.1)	5 (0.1)	41 (1.0)	486 (12.2)	0.43	1,353 (34.0)	0.13
45–64	**7,334 (29.1)**	5,046 (68.8)	517 (7.0)	58 (0.8)	158 (2.2)	1,555 (21.2)		2,814 (38.4)	
65–74	**5,813 (23.0)**	3,268 (56.2)	347 (6.0)	49 (0.8)	78 (1.3)	2,071 (35.6)		1,967 (33.8)	
75–84	**4,971 (19.7)**	2,490 (50.1)	249 (5.0)	36 (0.7)	63 (1.3)	2,133 (42.9)		1,621 (32.6)	
≥85	**3,150 (12.5)**	1,379 (43.8)	135 (4.3)	16 (0.5)	33 (1.0)	1,587 (50.4)		915 (29.0)	
**Sex**									
Male	**12,521 (49.6)**	7,767 (62.0)	778 (6.2)	81 (0.6)	178 (1.4)	3,717 (29.7)	0.05	4,489 (35.9)	0.07
Female	**12,720 (50.4)**	7,655 (60.2)	673 (5.3)	83 (0.7)	195 (1.5)	4,114 (32.3)		4,180 (32.9)	
Other/Unknown	**3 (—)**	2 (66.7)	0 (—)	0 (—)	0 (—)	1 (33.3)		1 (33.3)	
**Race/Ethnicity**									
White, non-Hispanic	**15,834 (62.7)**	9,288 (58.7)	910 (5.7)	94 (0.6)	229 (1.4)	5,313 (33.6)	0.22	5,061 (32.0)	0.16
Hispanic	**3,311 (13.1)**	2,200 (66.4)	200 (6.0)	24 (0.7)	48 (1.4)	839 (25.3)		1,344 (40.6)	
Black, non-Hispanic	**3,305 (13.1)**	2,386 (72.2)	200 (6.1)	18 (0.5)	44 (1.3)	657 (19.9)		1,299 (39.3)	
Other, non-Hispanic	**1,841 (7.3)**	906 (49.2)	95 (5.2)	24 (1.3)	37 (2.0)	779 (42.3)		608 (33.0)	
Unknown**	**953 (3.8)**	644 (67.6)	46 (4.8)	4 (0.4)	15 (1.6)	244 (25.6)		358 (37.6)	
**Underlying respiratory condition at discharge** ^††^									
Chronic respiratory condition	**14,842 (58.8)**	9,002 (60.7)	896 (6.0)	106 (0.7)	225 (1.5)	4,613 (31.1)	0.06	5,725 (38.6)	0.23
None	**10,402 (41.2)**	6,422 (61.7)	555 (5.3)	58 (0.6)	148 (1.4)	3,219 (30.9)		2,945 (28.3)	
**Underlying nonrespiratory condition at discharge^§§^**									
Chronic nonrespiratory condition	**22,131 (87.7)**	13,138 (59.4)	1,331 (6.0)	152 (0.7)	349 (1.6)	7,161 (32.4)	0.23	7,423 (33.5)	0.09
None	**3,113 (12.3)**	2,286 (73.4)	120 (3.9)	12 (0.4)	24 (0.8)	671 (21.6)		1,247 (40.1)	
**Any likely immunocompromise status** ^¶¶^									
Yes	**4,942 (19.6)**	2,636 (53.3)	330 (6.7)	40 (0.8)	103 (2.1)	1,833 (37.1)	0.18	1,346 (27.2)	0.16
No	**20,302 (80.4)**	12,788 (63.0)	1,121 (5.5)	124 (0.6)	270 (1.3)	5,999 (29.5)		7,324 (36.1)	
**No. of days from most recent dose to index date, median (IQR)**	**—**	—	264 (199–294)	52 (33–71)	48 (32–71)	59 (38–79)	—	—	—

**Abbreviations**: ICD-9 = *International Classification of Diseases, Ninth Revision*; ICD-10 = *International Classification of Diseases, Tenth Revision*; SMD = standardized mean or proportion difference.

* Medical events with a discharge code consistent with COVID-19–like illness were included. COVID-19–like illness diagnoses included acute respiratory illness (e.g., COVID-19, respiratory failure, or pneumonia) or related signs or symptoms (e.g., cough, fever, dyspnea, vomiting, or diarrhea) using ICD-9 and ICD-10 diagnosis codes. Clinician-ordered molecular assays (e.g., real-time reverse transcription–polymerase chain reaction) for SARS-CoV-2 infection occurring ≤14 days before to <72 hours after admission were included.

^†^ Vaccination status was categorized based on number and type of vaccine dose received before the medical event index date, which was the date of respiratory specimen collection associated with the most recent positive or negative SARS-CoV-2 test result before the medical event or the admission date if testing only occurred after admission. A primary Janssen vaccine dose was defined as 1 Janssen dose; a homologous booster dose following a primary Janssen dose was defined as 2 Janssen doses; a heterologous booster dose following a primary Janssen dose was defined as 1 Janssen/1 mRNA dose; a homologous booster dose following a primary mRNA series vaccination was defined as 3 mRNA doses.

^§^ Partners contributing data on medical events and estimated dates of Omicron variant predominance were in California (December 21), Colorado (December 19), Indiana (December 26), Minnesota and Wisconsin (December 25), New York (December 18), Oregon (December 24), Texas (December 16), Utah (December 24), and Washington (December 24).

**^¶^** An absolute SMD ≥0.20 indicates a nonnegligible difference in variable distributions between medical events for vaccinated versus unvaccinated patients and for positive versus negative test results. When calculating SMDs for differences in characteristics across COVID-19 vaccination status, investigators calculated the SMD as the average of the absolute value of the SMD for unvaccinated versus each vaccination status category individually (1 Janssen, 2 Janssen, 1 Janssen/1 mRNA, and 3 mRNA doses). All SMDs are reported as the absolute SMD.

** Unknown race/ethnicity includes Asian, Native Hawaiian or other Pacific islander, American Indian or Alaska Native, other not listed, and multiple races.

^††^ Underlying respiratory condition at discharge was defined as the presence of ICD-9 and ICD-10 discharge codes for asthma, chronic obstructive pulmonary disease, or other lung disease.

**^§§^** Underlying nonrespiratory condition at discharge was defined as the presence of ICD-9 and ICD-10 discharge codes for heart failure, ischemic heart disease, hypertension, other heart disease, stroke, other cerebrovascular disease, diabetes type I or II, other diabetes, metabolic disease, clinical obesity, clinically underweight, renal disease, liver disease, blood disorder, immunosuppression, organ transplant, cancer, dementia, neurologic disorder, musculoskeletal disorder, or Down syndrome.

^¶¶^ Immunocompromise status was defined as the presence of ICD-9 and ICD-10 discharge codes for solid malignancy, hematologic malignancy, rheumatologic or inflammatory disorder, other intrinsic immune condition or immunodeficiency, or organ or stem cell transplant.

Overall, VE against laboratory-confirmed COVID-19–associated hospitalization was significantly higher among patients who had received any booster dose (range = 67%–90%) compared with patients who had received 1 Janssen dose (31%) (Table 2). Among booster strategies, VE against hospitalizations was significantly higher among patients who had received 3 mRNA doses (90%). VE against hospitalizations was 78% after 1 Janssen/1 mRNA dose and 67% after 2 Janssen doses; however, CIs overlapped.

## Discussion

In a multivariate analysis of 80,287 ED/UC encounters and 25,244 hospitalizations among adults with COVID-19–like illness during Omicron variant predominance, VE for all booster strategies against ED/UC encounters and hospitalizations were higher than VE after 1 Janssen dose. Against ED/UC visits, the VE of a 1 Janssen/1 mRNA booster strategy was higher than that of 2 Janssen doses (79% versus 54%) and provided similar protection to 3 mRNA doses (2 primary mRNA doses followed by a homologous booster dose) (83%). Against hospitalizations, VE following 3 mRNA doses (90%) was higher than that following 1 Janssen/1 mRNA dose (78%) or 2 Janssen doses (67%).

The finding that a 1 Janssen/1 mRNA booster strategy had higher effectiveness than 2 Janssen doses against ED/UC visits and provided similar protection to 3 mRNA doses is consistent with data from a cohort study among U.S. veterans that indicated higher protection from 1 Janssen/1 mRNA dose against documented Omicron infection compared with 2 Janssen doses (*5*), as well as data from a recent test-negative design study that found both 1 Janssen/1 mRNA and 3 mRNA doses to have comparable effectiveness against symptomatic Omicron infection (CDC, unpublished data, 2022). This study adds to these findings by providing timely data on VE of different booster strategies against medically attended COVID-19–associated events from multiple health care systems and geographic regions of the U.S.

The findings in this report are subject to at least five limitations. First, among booster strategies, the median interval from receipt of most recent dose to medical event was 48–59 days and at most 120 days; thus, the observed effectiveness of these strategies is limited to a relatively short period after vaccination. Previous analysis within the VISION network identified waning of 3-mRNA–dose VE with increasing time since vaccination (*6*); continual investigations on the durability of protection provided by different booster strategies are warranted. Second, the small number of Janssen vaccine recipients reduced the precision of VE estimates across both primary series and booster strategy groups. The small number of recipients also precluded estimation of VE stratified by demographic factors including age and race, or assessment for potential effect modification due to underlying conditions, including immunocompromise; however, sensitivity analysis limited to immunocompetent persons found no significant change in results. Third, although adjustments to account for differences between unvaccinated and vaccinated persons were made, they did not account for differences among persons vaccinated with different strategies. In addition, residual bias might exist from misclassification or incomplete ascertainment of data on the presence of immunocompromise, other health conditions, vaccination status, and unmeasured behaviors (e.g., mask use and close contact with persons with COVID-19). Fourth, genetic characterization of viral variants causing infection among patients was not available, and analyses relied on dates when the Omicron variant became locally predominant based on surveillance data; therefore, the early phase of Omicron variant predominance in this study likely includes some medical encounters associated with the B.1.617.2 (Delta) variant. Finally, although the facilities in this study serve heterogeneous populations in 10 states, the findings might not be generalizable to the entire U.S. population.

These findings underscore the importance of receiving recommended COVID-19 booster doses, when eligible, to prevent moderate to severe COVID-19 during Omicron variant predominance. All adults who have received mRNA vaccines for their COVID-19 primary series vaccination should receive an mRNA booster dose when they are eligible. Adults who received a Janssen vaccine as their first dose should preferentially receive a heterologous mRNA vaccine booster dose ≥2 months later, or a homologous Janssen vaccine booster dose if mRNA vaccine is contraindicated or unavailable.

**Summary**

**What is already known about this topic?**

Little is known about vaccine effectiveness (VE) of different booster strategies following Ad.26.COV2.S (Janssen [Johnson & Johnson]) vaccination, especially during Omicron variant predominance.

**What is added by this report?**

VE against COVID-19–associated emergency department/urgent care visits was 24% after 1 Jansen dose, 54% after 2 Jansen doses, and 79% after 1 Janssen/1 mRNA dose, compared to 83% after 3 mRNA doses. VE for the same strategies against COVID-19–associated hospitalization was 31%, 67%, 78%, and 90% respectively.

**What are the implications for public health practice?**

All eligible persons should receive recommended COVID-19 booster doses to prevent moderate to severe COVID-19. Adult Janssen primary vaccine recipients should preferentially receive a heterologous mRNA vaccine booster dose ≥2 months later.

## Footnotes

* These authors contributed equally to this report.

† On October 15, 2021, the Food and Drug Administration (FDA) authorized a single Janssen COVID-19 vaccine booster dose in persons aged ≥18 years who received a Janssen COVID-19 vaccine dose ≥2 months earlier. On October 20, 2021, FDA released an amendment allowing for heterologous boosting of all currently authorized COVID-19 vaccines (BNT162b2 [Pfizer-BioNTech], mRNA-1273 [Moderna], and Janssen) (https://www.fda.gov/media/153441/download). On October 21, 2021, CDC recommended that adults aged ≥18 years who received a Janssen COVID-19 vaccine should receive a single COVID-19 vaccine booster dose ≥2 months later (https://www.cdc.gov/media/releases/2021/p1021-covid-booster.html). On December 16, 2021, following an updated benefit-risk assessment which accounted for risks of thrombosis with thrombocytopenia syndrome and Guillain-Barré syndrome following receipt of a Janssen vaccine, CDC recommended preferential use of mRNA COVID-19 vaccines over the Janssen COVID-19 vaccine, for both primary and booster doses among adults aged ≥18 years (https://www.cdc.gov/media/releases/2021/s1216-covid-19-vaccines.html). Current COVID-19 vaccine booster dose recommendations are available at https://www.cdc.gov/vaccines/covid-19/clinical-considerations/covid-19-vaccines-us.html.

§ The VISION Network includes Baylor Scott & White Healthcare (Texas), Columbia University Irving Medical Center (New York), HealthPartners (Minnesota and Wisconsin), Intermountain Healthcare (Utah), Kaiser Permanente Northern California (California), Kaiser Permanente Northwest (Oregon and Washington), Regenstrief Institute (Indiana), and University of Colorado (Colorado).

¶ ED data at Columbia University Irving Medical Center and HealthPartners exclude encounters that were transferred to an in-network hospital.

** Partners contributing data on medical events (and estimated dates of Omicron predominance) were in California (December 21), Colorado (December 19), Indiana (December 26), Minnesota and Wisconsin (December 25), New York (December 18), Oregon (December 24), Texas (December 16), Utah (December 24), and Washington (December 24).

†† COVID-19–like illness diagnoses included acute respiratory illness (e.g., COVID-19, respiratory failure, or pneumonia) or related signs or symptoms (e.g., cough, fever, dyspnea, vomiting, or diarrhea) using diagnosis codes from the *International Classification of Diseases, Ninth Revision* and *International Classification of Diseases, Tenth Revision*.

§§ Both Pfizer-BioNTech and Moderna COVID-19 vaccines were included in mRNA vaccine categories. Among eligible ED/UC encounters and hospitalizations, among recipients of 1 Janssen/1 mRNA dose, 48% had received a Pfizer-BioNTech mRNA booster dose, and 52% had received a Moderna booster dose. Among recipients of 3 mRNA doses, 42% had received all Pfizer-BioNTech mRNA vaccines, 49% had received all Moderna vaccines, and 9% had received a mix of mRNA vaccine doses.

¶¶ The index date for each medical visit was defined as either the date of collection of a respiratory specimen associated with the most recent positive or negative SARS-CoV-2 test result before the medical visit or the date of the medical visit (if testing occurred only after the admission or visit date).

*** Among 84,813 eligible ED/UC encounters, 4,526 (5.3%) were removed based on these exclusion criteria. Among 27,308 eligible hospitalizations, 2,064 (7.6%) were removed. The third mentioned exclusion criterion would remove persons who were moderately or severely immunocompromised and had received a second mRNA dose 4 weeks after a primary Janssen vaccine dose or a third mRNA dose 4 weeks after a second dose as part of a primary mRNA series. https://www.cdc.gov/vaccines/covid-19/clinical-considerations/covid-19-vaccines-us.html#vaccination-people-immunocompromised

††† Immunocompromising conditions were derived from lists used in previous studies of large hospital-based or administrative databases and included 1) solid malignancies, 2) hematologic malignancies, 3) rheumatologic or inflammatory disorders, 4) other intrinsic immune conditions or immunodeficiencies, and 5) organ or stem cell transplants.

§§§ With a test-negative design, vaccine performance is assessed by comparing the odds of antecedent vaccination among case-patients with acute laboratory-confirmed COVID-19 and control-patients without acute laboratory-confirmed COVID-19. VE was calculated as [1 − odds ratio] x 100%. Generalized boosted regression trees were used to estimate the propensity to be vaccinated based on sociodemographic characteristics, underlying medical conditions, and patient and facility characteristics. Of the variables included in the propensity score, previous SARS-CoV-2 testing and test positivity were not balanced after applying inverse propensity weights and thus were added to covariates included in the adjusted VE model.

¶¶¶ 45 C.F.R. part 46; 21 C.F.R. part 56.
